# Intramedullary Femoral Nailing—Blade Versus Screw: A Risk Analysis of Implant-Related Complications in Trochanteric Femoral Fractures

**DOI:** 10.3390/jcm15187221

**Published:** 2026-09-17

**Authors:** Theodoros Loupasis, Method Kabelitz, Nikolaos Trygonis, Adriano Fabi, Manuel Waltenspül, Michael Dietrich

**Affiliations:** 1 Clinic for Orthopaedics, Hand and Trauma Surgery, Stadtspital Zürich, 8037 Zurich, Switzerland; theodoros.loupasis@stadtspital.ch (T.L.); nikolas.trygonis@stadtspital.ch (N.T.); manuel.waltenspuel@stadtspital.ch (M.W.); michael.dietrich@stadtspital.ch (M.D.); 2 Faculty of Health Sciences & Medicine, University of Lucerne, Frohburgstrasse 3, 6002 Luzern, Switzerland; 3 Department of Orthopaedics and Trauma, Lucerne Cantonal Hospital (LUKS), 6000 Luzern, Switzerland; adriano.fabi@luks.ch; 4 Department of Plastic, Reconstructive, Aesthetic and Hand Surgery, University Hospital Basel, 4031 Basel, Switzerland

**Keywords:** trochanteric fracture, cephalomedullary nailing, fragility fracture, complications, Trochanteric Femoral Nail Advanced, helical blade, lag screw, reduction quality, surgical revision

## Abstract

**Background/Objectives**: Previous studies comparing helical blade and lag screw fixation have often compared different cephalomedullary nail systems, leaving the specific association of the cephalic fixation element with clinical outcomes unclear. This study compared implant-related complications after fixation with the Trochanteric Femoral Nail Advanced (TFNA) using either a helical blade or a lag screw. **Methods**: A retrospective single-center cohort study included consecutive patients treated with a TFNA for AO/OTA 31-A1 to 31-A3 fractures between January 2023 and December 2025. Mechanical complications, surgical revisions, revision-free survival and radiographic risk factors were evaluated using univariable and adjusted analyses. **Results**: A total of 277 patients were included (136 helical blade, 141 lag screw). Overall postoperative complications were comparable between groups (23.5% vs. 18.4%, *p* = 0.306). Surgical revision occurred significantly more frequently following helical blade fixation (22.1% vs. 12.1%, *p* = 0.037), remaining significant after adjustment (adjusted RR 1.84, 95% CI 1.07–3.15; *p* = 0.027). However, this association was not statistically significant in time-to-event analyses or in a sensitivity analysis additionally adjusting for continuous calendar time (screw vs. blade: adjusted RR 0.56, 95% CI 0.19–1.72; *p* = 0.313). Cut-in occurred exclusively after blade fixation (4.4% vs. 0.0%), although this exploratory difference was not statistically significant after correction for multiple comparisons, whereas conversion to total hip arthroplasty (10.3% vs. 5.0%, *p* = 0.114) and revision-free survival did not differ significantly. Poor Baumgaertner reduction quality was associated with postoperative complications (*p* = 0.004) and mechanical failure (*p* = 0.003), while selected measures of residual calcar and fragment displacement were also associated with adverse outcomes. Fracture classification, cortical thickness index, tip–apex distance and postoperative alignment were not associated with surgical revision (all *p* > 0.05). **Conclusions**: Within the TFNA system, helical blade fixation was associated with a higher observed risk of surgical revision in the primary analysis; however, this association was not confirmed in time-to-event or calendar-time-adjusted analyses. Reduction quality and residual fracture displacement remained key determinants of adverse outcomes, emphasizing the importance of meticulous fracture reduction.

## 1. Introduction

Trochanteric fractures of the proximal femur represent a clinical challenge, particularly in the growing population of geriatric patients with osteoporotic bone [1]. Early operative stabilization remains crucial to facilitate mobilization and reduce morbidity. Cephalomedullary nailing has become the standard treatment modality due to its biomechanical advantages in managing unstable fracture patterns. Despite these benefits, mechanical complications in the femoral head and neck—including implant cut-out, cut-through and cut-in—remain a substantial problem, with reported revision rates of up to 20% in selected high-risk fracture configurations [2,3]. Bone quality, fracture morphology according to the Arbeitsgemeinschaft für Osteosynthesefragen/Orthopaedic Trauma Association (AO/OTA) classification [4], and implant positioning within the femoral head have long been recognized as key determinants of mechanical stability, as originally conceptualized by Kaufer et al. [5].

Contemporary cephalomedullary nails offer different options for femoral head–neck fixation, most commonly a helical blade or a conventional lag screw. Biomechanically, the helical blade compacts cancellous bone during insertion, potentially improving fixation in osteoporotic bone, whereas lag screw insertion requires drilling and removes more cancellous bone [6]. Despite these theoretical advantages, clinical evidence comparing helical blades and lag screws remains conflicting. Stern et al. reported higher cut-out rates associated with helical blades compared with lag screws in earlier-generation devices [7]. In contrast, a recent meta-analysis found no association between cephalomedullary implant type and cut-out risk, while confirming that a tip–apex distance (TAD) greater than 25 mm is a robust predictor of mechanical failure [8]. These conflicting findings suggest that implant choice alone may not fully explain mechanical failure. Instead, fracture morphology, reduction quality, implant positioning and bone quality may substantially influence clinical outcomes. However, the relative contribution of these factors compared with implant design remains incompletely understood.

An important limitation of the existing evidence is that many previous comparisons of helical blade and lag screw fixation involved different cephalomedullary nail systems, making it difficult to isolate the association of the cephalic fixation element itself with clinical outcomes. TFNA-specific comparative data using the Trochanteric Femoral Nail Advanced (TFNA; DePuy Synthes, Zuchwil, Switzerland) and incorporating detailed postoperative reduction parameters also remain limited. Therefore, this retrospective study compared implant-related complications and surgical revision between TFNA helical blade and lag screw fixation within the same cephalomedullary nail platform and evaluated the associations of clinical and radiographic factors—including reduction quality, fracture morphology, implant positioning and bone quality—with postoperative complications and mechanical failure. By isolating the cephalic fixation element within a single TFNA system, this study aimed to better distinguish implant-related associations from potentially modifiable technical factors.

## 2. Materials and Methods

### 2.1. Study Design and Patient Selection

**Figure 1:**
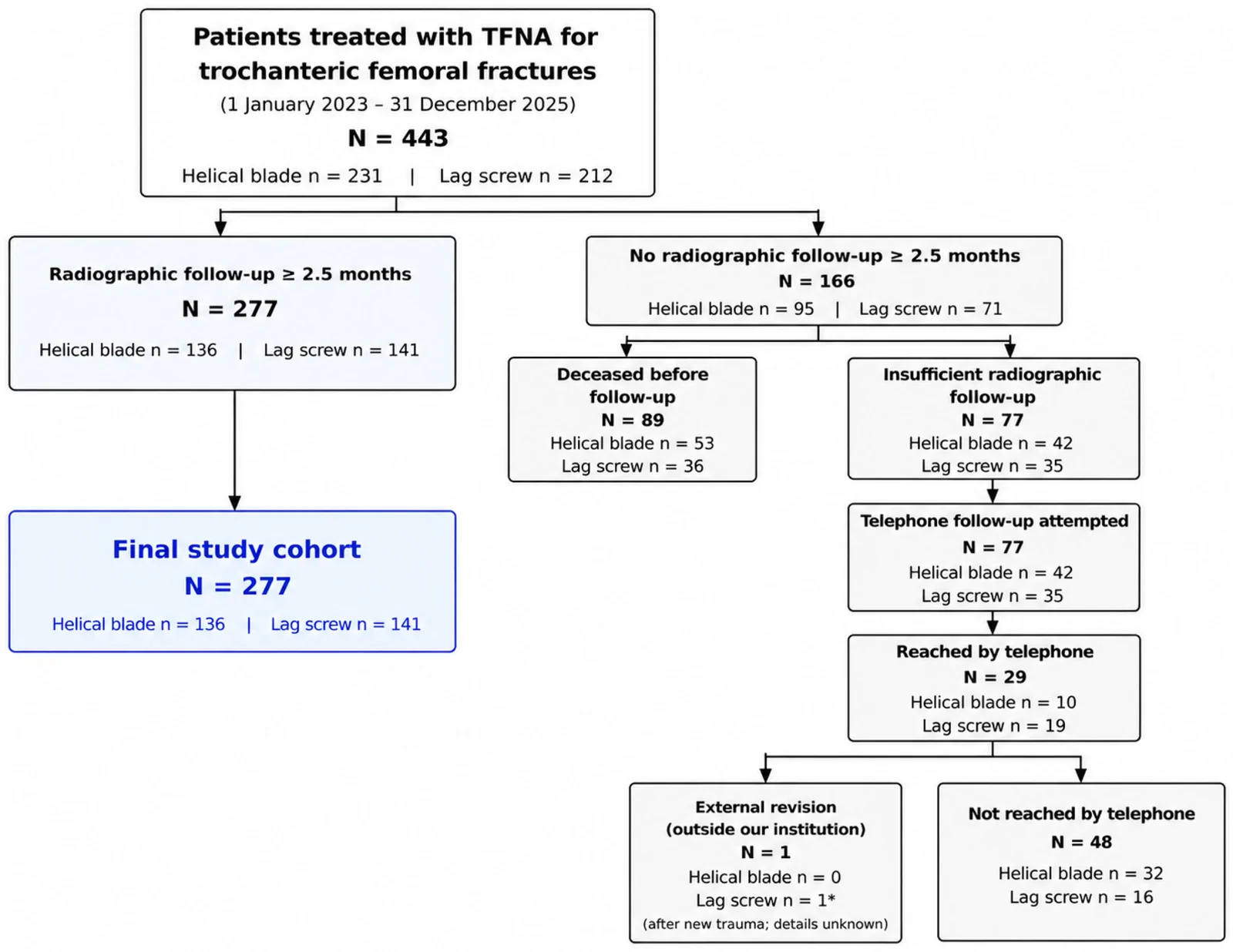
Flowchart of patient selection. Consecutive patients treated with a TFNA for AO/OTA 31-A1 to 31-A3 trochanteric femoral fractures between January 2023 and December 2025 were screened for eligibility. Patients with radiographic follow-up ≥ 2.5 months constituted the primary study cohort. Patients without sufficient radiographic follow-up underwent additional chart review and telephone follow-up, where possible, to identify revisions performed outside our institution. The threshold of 2.5 months was chosen to capture the routine postoperative radiographic assessment performed approximately 3 months after surgery while allowing standardized identification of eligible patients within the electronic database. TFNA, Trochanteric Femoral Nail Advanced. * Helical blade converted to lag screw externally following a new accident.

This retrospective, non-interventional cohort study was conducted at a Level I trauma center and is reported in accordance with the Strengthening the Reporting of Observational Studies in Epidemiology (STROBE) statement for cohort studies. Consecutive patients who underwent operative treatment with a Trochanteric Femoral Nail Advanced (TFNA; DePuy Synthes, Zuchwil, Switzerland) for AO/OTA 31-A1 to 31-A3 trochanteric femoral fractures between 1 January 2023 and 31 December 2025 were screened for eligibility. Patient selection is summarized in Figure 1.

Implant selection was determined exclusively by the institutional standard of care rather than surgeon preference. Until June 2024, the TFNA helical blade was routinely used. During a transition period in the third quarter of 2024, both helical blade and lag screw fixation were used, after which the TFNA lag screw became the institutional standard implant for all subsequent cases. All procedures were performed according to the institutional standard of care by consultant orthopaedic trauma surgeons with resident assistance. Of 443 consecutive patients (231 helical blade and 212 lag screw), 277 met the predefined inclusion criterion of radiographic follow-up ≥ 2.5 months and constituted the primary study cohort (136 helical blade and 141 lag screw). Radiographic follow-up of at least 2.5 months corresponded to the institution’s routine postoperative radiographic assessment performed approximately 3 months after surgery. The 2.5-month threshold was chosen to allow standardized identification of eligible patients within the electronic database. Patients without sufficient radiographic follow-up underwent additional chart review and telephone follow-up, where possible, to identify revisions performed outside our institution. Patients were eligible if they were aged ≥ 18 years, sustained an AO/OTA 31-A1 to 31-A3 trochanteric femoral fracture, were treated with a TFNA using either a helical blade or a lag screw, and had radiographic follow-up of at least 2.5 months. Patients were eligible for inclusion only if complete clinical and radiographic data were available. Patients with previous surgery or revision of the affected hip, periprosthetic or peri-implant fractures, pathological fractures, documented objection to data use, or immediate postoperative death precluding radiographic assessment were not eligible for inclusion. The study was approved by the regional ethics committee, and all data were pseudonymized prior to analysis in accordance with applicable data protection regulations.

### 2.2. Data Collection and Outcome Definitions

Data were retrospectively extracted from the institutional electronic medical record and radiographic archive. Predefined demographic, clinical, operative and radiographic variables were extracted, including age, sex, body mass index (BMI), American Society of Anesthesiologists (ASA) physical status classification, cognitive impairment at admission, pre-injury mobility, use of anti-osteoporotic medication at admission, comorbidities, use of a helical blade or lag screw, cement augmentation, cerclage use, intraoperative complications, AO/OTA fracture classification, tip–apex distance (TAD), femoral cortical thickness index (CTI) as an indirect measure of bone quality, and initial postoperative alignment.

The primary outcome was surgical revision due to implant-related complications. Secondary outcomes included overall postoperative complications, defined as cut-out, cut-in, cut-through, lateralization of the head–neck fixation element, non-union, peri-implant fracture, implant breakage and peri-implant infection. An exploratory analysis evaluated postoperative mechanical failure, defined as cut-out, cut-in and cut-through. All suspected mechanical failures were independently assessed by two investigators (T.L. and N.T.) and confirmed by a senior orthopaedic trauma surgeon (M.K.). Discrepancies were resolved by consensus. Associations between surgical revision, postoperative complications, mechanical failure and radiographic and clinical risk factors—including fracture morphology, postoperative alignment, TAD, CTI, anti-osteoporotic medication at admission, and the type of head–neck fixation (helical blade versus lag screw)—were also investigated.

### 2.3. Radiographic Assessment

Radiographic assessment was performed using standardized postoperative anteroposterior and lateral radiographs retrieved from the institutional picture archiving and communication system (PACS). Fractures were classified according to the AO/OTA classification [4]. TAD, CTI and postoperative alignment (neutral, varus or valgus) were measured retrospectively using the institutional PACS according to previously published methods [9]. Specifically, TAD was measured according to Baumgaertner et al., while CTI and postoperative alignment were assessed according to their respective published methods. Radiographic measurements were performed by three investigators (T.L., M.K. and N.T.), including two board-certified orthopaedic trauma surgeons and one orthopaedic surgery resident.

### 2.4. Statistical Analysis

Statistical analyses were performed in R (version 4.3.2; R Foundation for Statistical Computing, Vienna, Austria). Distributional assumptions for continuous variables were assessed using the Shapiro–Wilk test. Age and BMI showed statistically significant deviations from normality in both implant groups; however, given the sample size of each group, between-group comparisons were performed using Welch’s independent-samples *t*-test, which is robust to moderate deviations from normality. Age and BMI are therefore reported as mean ± standard deviation. Time from injury to surgery showed a more markedly skewed distribution and was reported as median with interquartile range (IQR) and compared using the Wilcoxon rank-sum test. Categorical variables were presented as numbers and percentages and compared using Fisher’s exact test. For categorical variables with more than two levels, a global Fisher’s exact test was used to compare the overall distribution between groups. Adjusted risk ratios (RRs) with 95% confidence intervals (CIs) were estimated using modified Poisson regression with robust standard errors. For the patient-characteristic analyses, implant type, age, sex, and osteoporosis were entered simultaneously into each model. For the radiographic analyses, each radiographic predictor was evaluated in a separate model adjusted for implant type, age, and sex in order to limit model complexity and avoid simultaneous inclusion of closely related radiographic measures. The additional sensitivity analysis of postoperative alignment comprised three multivariable models: a core model including implant type, age, sex, AO/OTA classification, fracture-side CTI, contralateral CTI, TAD, and tip position; a reduction-component model additionally including calcar displacement, fragment displacement, and angulation; and a Baumgaertner model in which Baumgaertner reduction quality was included instead of the individual reduction components. Missing data were handled using complete-case analysis within each model, without imputation. Multicollinearity was assessed using variance inflation factors in the reduction-component model. Variance inflation factors ranged from 1.14 to 2.10, indicating no evidence of problematic multicollinearity among variables retained in the reduction-component model. Given the limited number of surgical revision and mechanical failure events, analyses of radiographic predictors and the additional alignment sensitivity analyses were considered exploratory and hypothesis-generating, and model complexity was limited where possible. To assess potential temporal confounding related to the institutional change in implant use, an additional sensitivity analysis of surgical revision was performed using modified Poisson regression including implant type, continuous calendar time, age, and sex. Revision-free survival was estimated using Kaplan–Meier curves, with any surgical revision defined as the event of interest, and compared between groups using the log-rank test. As a sensitivity analysis addressing the unequal follow-up duration between implant groups, revision-free survival was additionally estimated at fixed postoperative time points of 90 and 180 days. Restricted mean survival time (RMST) up to 90 days was also compared between groups. Additionally, a Cox proportional hazards regression model was used to evaluate the association between implant type and revision-free survival, with adjustment for age, sex, and osteoporosis, and to estimate hazard ratios (HRs) with 95% confidence intervals. For exploratory comparisons of individual postoperative complication subtypes, Holm and Benjamini–Hochberg corrections were applied as post hoc sensitivity analyses. All statistical tests were two-sided, and a *p*-value of <0.05 was considered statistically significant.

## 3. Results

### 3.1. Patient Characteristics

A total of 277 patients met the inclusion criteria and were included in the final analysis, comprising 136 patients treated with a TFNA helical blade and 141 treated with a TFNA lag screw. Baseline demographic and clinical characteristics are presented in Table 1. The mean age of the overall cohort was 80.6 ± 10.7 years, and 70.8% of patients were female. The two treatment groups were well balanced, with no statistically significant differences in demographic or clinical baseline characteristics (all *p* > 0.05).

### 3.2. Fracture and Operative Characteristics

Overall, fracture characteristics, operative variables and postoperative radiographic parameters were well balanced between the helical blade and lag screw groups (Table 2). Significant differences were observed only for drainage use, which was more frequent in the blade group (14.1% vs. 1.4%, *p* < 0.001), and for blade/screw tip position (*p* = 0.044), with central tip placement occurring more frequently after helical blade fixation. All remaining variables, including AO/OTA fracture classification, cortical thickness index, tip–apex distance, postoperative alignment, calcar displacement, fragment displacement, fracture angulation and Baumgaertner reduction quality, were comparable between groups (all *p* > 0.05).

### 3.3. Complications and Revisions

#### 3.3.1. Overall Complications

Postoperative complications occurred in 58 patients (20.9%) and were numerically more frequent in the helical blade group than in the lag screw group (23.5% vs. 18.4%), although this difference was not statistically significant (*p* = 0.306). Overall surgical revision was performed significantly more often after helical blade fixation than after lag screw fixation (22.1% vs. 12.1%, *p* = 0.037) (Table 3).

#### 3.3.2. Specific Complications

In exploratory analyses of individual postoperative complications, cut-in occurred exclusively in the helical blade group (4.4% vs. 0.0%, unadjusted *p* = 0.013). However, this difference was no longer statistically significant after correction for multiple comparisons (Holm-adjusted *p* = 0.146; Benjamini–Hochberg-adjusted *p* = 0.146). Cut-out was more frequent following helical blade fixation (11.0% vs. 5.0%; unadjusted *p* = 0.076), but this difference was not statistically significant. No statistically significant between-group differences were observed for cut-through, peri-implant fracture, infection, non-union, implant breakage or other individual postoperative complications.

#### 3.3.3. Revision Procedures

Revision to a lag screw was performed more frequently in patients initially treated with a helical blade (6.6% vs. 0.7%, *p* = 0.009). Rates of conversion to total hip arthroplasty and all other revision procedures were comparable between groups.

### 3.4. Adjusted Analyses

Adjusted analyses demonstrated that helical blade fixation remained associated with a higher risk of surgical revision after adjustment for age, sex, and osteoporosis (adjusted RR 1.84, 95% CI 1.07–3.15; *p* = 0.027). In contrast, implant type was not independently associated with either intraoperative complications (adjusted RR 1.10, 95% CI 0.29–4.22; *p* = 0.884) or overall postoperative complications (adjusted RR 1.27, 95% CI 0.80–2.01; *p* = 0.306). None of the covariates included in the adjusted models—including age, sex and osteoporosis—were independently associated with intraoperative complications, postoperative complications or surgical revision.

To identify fracture- and surgery-related predictors of adverse outcomes, additional adjusted analyses were performed (Table 4). Neither fracture classification, cortical thickness index, tip–apex distance nor postoperative alignment was independently associated with postoperative complications or surgical revision after adjustment for implant type, age and sex.

In contrast, several postoperative radiographic parameters were associated with postoperative complications after adjustment for implant type, age, and sex. Medial and lateral calcar displacement on the anteroposterior radiograph, anterior calcar displacement on the lateral radiograph, fragment displacement greater than 4 mm on the anteroposterior radiograph, and poor reduction quality were all associated with a significantly increased risk of postoperative complications.

Because implant type was strongly associated with treatment period, an additional sensitivity analysis incorporating continuous calendar time was performed. The direction of the implant-type association remained consistent with the primary analysis, although the estimate was imprecise and not statistically significant (screw vs. blade: adjusted RR 0.56, 95% CI 0.19–1.72; *p* = 0.313). To further characterize factors associated with implant-related mechanical failure, an exploratory analysis was performed (Table 5). No association with mechanical failure was demonstrated for fracture classification, cortical thickness index, tip–apex distance, or blade/screw tip position. In contrast, valgus alignment, calcar displacement, fragment displacement greater than 4 mm, and poorer Baumgaertner reduction quality were associated with an increased risk of mechanical failure after adjustment for implant type, age, and sex. Given the limited number of mechanical failure events, these findings should be regarded as hypothesis-generating.

### 3.5. Sensitivity Analysis of Postoperative Alignment and Mechanical Failure

Because the association between valgus alignment and postoperative mechanical failure appeared biologically unexpected, additional sensitivity analyses were performed to evaluate whether this finding was explained by postoperative reduction quality (Table 6). In the core adjusted model, valgus alignment remained associated with a higher risk of mechanical failure. However, after additional adjustment for postoperative reduction-quality parameters, including calcar displacement, fragment displacement and angulation, this association was markedly attenuated and no longer statistically significant. Similar findings were observed when Baumgaertner reduction quality was included instead of the individual reduction components. These exploratory findings suggest that the observed association between valgus alignment and mechanical failure may partly reflect accompanying abnormalities in overall reduction quality rather than an effect of alignment alone.

### 3.6. Adjusted Cox Proportional Hazards Analysis

To account for differences in follow-up duration between treatment groups, a Cox proportional hazards model was performed with implant type as the exposure of interest and adjustment for age, sex, and osteoporosis (Table 7). Helical blade fixation was associated with a numerically higher hazard of revision than lag screw fixation, although this association did not reach statistical significance (HR 1.46, 95% CI 0.78–2.73; *p* = 0.240). Age, sex, and osteoporosis were likewise not significantly associated with revision-free survival.

### 3.7. Kaplan–Meier Analysis of Revision-Free Survival

**Figure 2:**
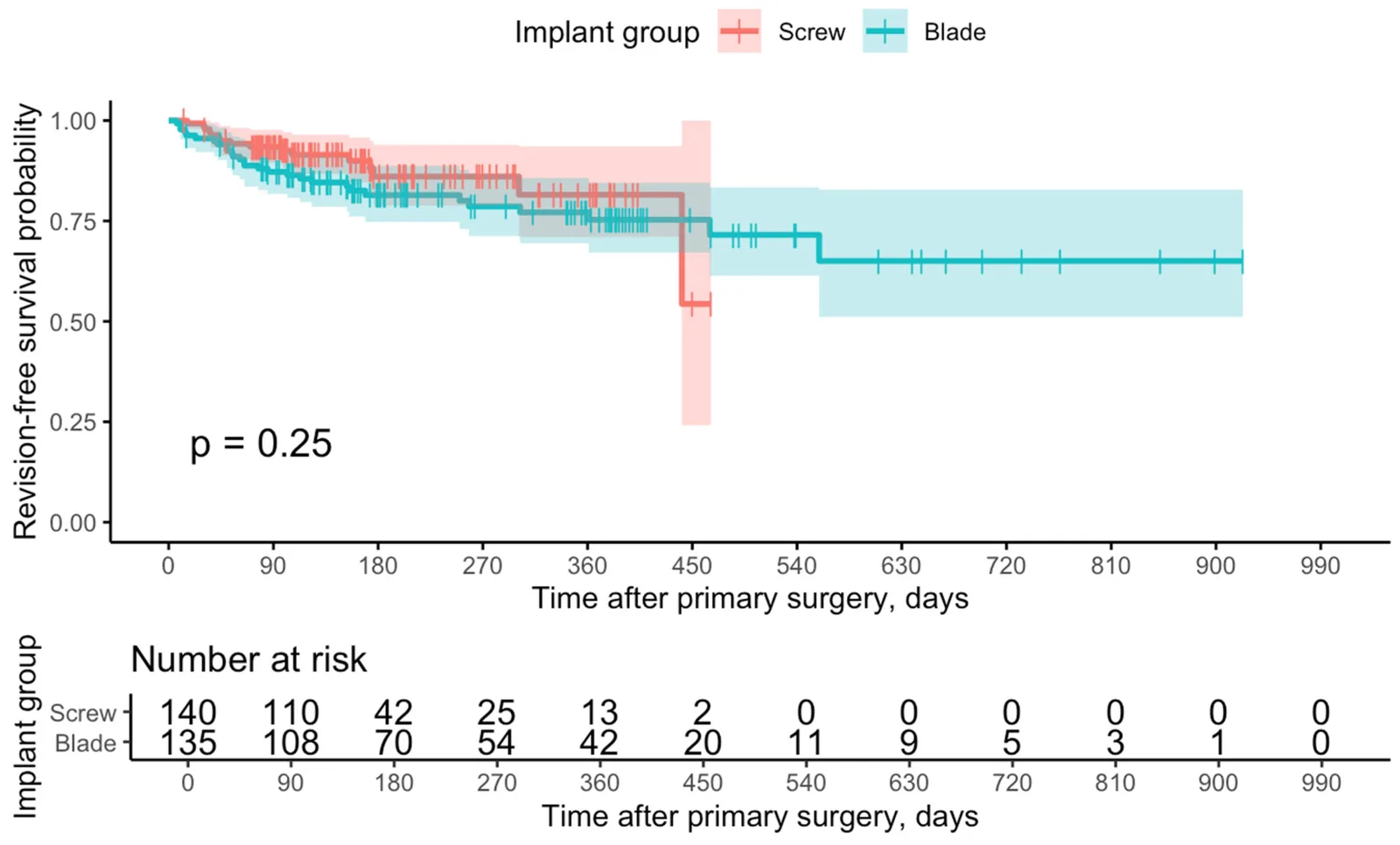
Kaplan–Meier estimates of revision-free survival according to implant group. Curves show revision-free survival after the primary procedure in the blade and screw groups. Shaded areas represent 95% confidence intervals. Groups were compared using the log-rank test.

The mean follow-up duration was significantly longer in the helical blade group than in the lag screw group (299.2 ± 203.5 vs. 165.2 ± 100.9 days; *p* < 0.001) (Table 8). To account for this difference in follow-up duration, revision-free survival was additionally evaluated using Kaplan–Meier analysis (Figure 2). Two patients with missing follow-up information were excluded from the survival analysis. Kaplan–Meier analysis showed no statistically significant difference in revision-free survival between implant groups (log-rank *p* = 0.25). Although the lag screw group demonstrated numerically fewer revisions, this difference did not reach statistical significance. Fixed-time estimates showed the same directional pattern. Revision-free survival at 90 days was 87.2% (95% CI 81.7–93.1%) in the blade group and 93.5% (95% CI 89.5–97.7%) in the screw group. At 180 days, the corresponding estimates were 81.4% (95% CI 74.8–88.7%) and 86.1% (95% CI 78.8–94.0%), respectively. The 90-day RMST difference between the blade and screw groups was −2.8 days (95% CI −6.5 to 1.0; *p* = 0.147), with an RMST ratio of 0.97 (95% CI 0.93–1.01; *p* = 0.149).

## 4. Discussion

The present study compared clinical and radiographic outcomes following TFNA fixation using a helical blade or a lag screw in patients with trochanteric femoral fractures. Four principal findings emerged. First, helical blade fixation was associated with a higher risk of surgical revision in the primary analysis, whereas no association was observed for intraoperative or overall postoperative complications. Second, postoperative radiographic reduction parameters—including calcar displacement, fragment displacement and poor Baumgaertner reduction quality—were stronger predictors of postoperative complications and mechanical failure than fracture classification, cortical thickness index, tip–apex distance or postoperative alignment. Third, poor Baumgaertner reduction was associated with postoperative complications, surgical revision, and mechanical failure. Finally, although revision-free survival tended to be better following lag screw fixation, this difference did not reach statistical significance.

Our study demonstrated that patients treated with a TFNA helical blade had a significantly higher risk of surgical revision than those treated with a lag screw after adjustment for age, sex, and osteoporosis. In contrast, implant type was not associated with intraoperative or general postoperative complications, suggesting that the observed effect was specific to mechanical implant failure rather than perioperative morbidity.

The current literature remains divided regarding the influence of cephalic implant design on fixation failure. A recent meta-analysis by Kim et al. [10] reported a higher overall risk of fixation failure with helical blades, which was primarily driven by an increased incidence of cut-through rather than conventional cut-out. In contrast, the meta-analysis by Ng et al. [8] found no significant difference in cut-out rates between blades and lag screws, identifying reduction quality and implant positioning as the dominant determinants of failure. Similarly, randomized studies have generally failed to demonstrate clinically relevant differences between implant designs, although most compared different nail systems rather than isolating the cephalic fixation element itself [11,12,13].

Our findings raise the hypothesis that blade fixation may be associated with a distinct failure pattern. In exploratory analyses, cut-in occurred exclusively in the blade group, whereas conventional cut-out was observed in both groups. However, this difference was no longer statistically significant after correction for multiple comparisons and should therefore be regarded as hypothesis-generating. The observed pattern is nevertheless consistent with previous reports describing medial migration, or atypical medial cut-out, as a blade-specific complication [14]. However, our results should be interpreted cautiously. The largest registry study to date, including more than 22,000 patients, reported no overall difference in aseptic revision rates between blades and lag screws [11], while the only contemporary TFNA-specific comparative study likewise found no independent effect of implant type [13]. Both studies, however, were limited by very low numbers of mechanical failures, and the TFNA cohort included only five fixation failures during six months of follow-up.

An important strength of the present study is that both treatment groups received the same cephalomedullary nail, differing only in the cephalic fixation element. Many previous comparisons evaluated different nail generations or implant systems simultaneously, making it difficult to distinguish the effect of the blade or lag screw from differences in nail geometry, sliding characteristics or implant design. By isolating the cephalic fixation element within a single TFNA platform, our findings provide more specific evidence regarding the potential contribution of implant design to mechanical failure. Nevertheless, given the retrospective design and limited number of revision events, our results should be interpreted as demonstrating an association rather than establishing causality.

In the present study, postoperative reduction quality emerged as one of the strongest determinants of mechanical outcome. Poor reduction according to the Baumgaertner criteria, residual calcar displacement and postoperative fragment displacement were independently associated with postoperative complications and mechanical failure, whereas fracture classification, cortical thickness index and tip–apex distance were not independent predictors.

These findings are consistent with growing evidence suggesting that the quality of fracture reduction is more important than the initial fracture morphology. Ibrahim et al. [15] identified poor reduction quality as the strongest independent predictor of fixation failure, whereas implant type was not associated with cut-out after multivariable adjustment. Likewise, the recent meta-analysis by Ng et al. [8] demonstrated that inadequate reduction and implant positioning were consistently associated with fixation failure, highlighting reduction quality as one of the few modifiable surgical risk factors. Most recently, Ho et al. [12] and Wittauer et al. [13] reported that postoperative reduction quality, rather than implant type, fracture classification or TAD, independently predicted fixation failure following TFNA fixation.

The observed association between calcar and fragment displacement is also biomechanically plausible. Restoration of anteromedial cortical support has been recognized as a key determinant of construct stability, allowing controlled fracture impaction while preventing excessive varus collapse and secondary displacement. Chang et al. [16] first introduced the concept of positive medial cortical support, demonstrating improved maintenance of reduction and better clinical outcomes. Subsequently, Song et al. [17] showed that residual calcar gaps on both anteroposterior and lateral radiographs independently predicted loss of anteromedial cortical support during follow-up. Similarly, Fan et al. [18] identified poor reduction quality and loss of medial support as independent predictors of implant failure. These findings closely parallel our observation that residual calcar and fragment displacement were stronger predictors of adverse outcomes than the original fracture pattern itself.

Interestingly, AO/OTA fracture classification was not independently associated with mechanical failure in our cohort. This finding supports the concept that fracture morphology primarily reflects the initial injury pattern, whereas postoperative reduction better represents the final biomechanical environment after fixation. Similarly, no independent association between CTI and adverse outcomes was demonstrated in the present cohort. This finding should not be interpreted as evidence that bone quality is clinically unimportant. CTI represents an indirect radiographic surrogate of bone quality and may be influenced by femoral anatomy and radiographic projection; it does not provide the same information as direct assessment of bone mineral density. Furthermore, the limited number of mechanical failure events may have reduced the statistical power to detect such an association.

We did not demonstrate an independent association between TAD and mechanical failure in the present cohort. Although TAD remains an established predictor of conventional cut-out, particularly when exceeding 25 mm, its predictive value may be reduced in contemporary TFNA cohorts with generally acceptable implant positioning and when composite mechanical endpoints include blade-specific failure mechanisms such as cut-in or cut-through. Our findings therefore should not be interpreted as questioning the clinical importance of TAD but rather suggest that residual fracture instability may provide additional prognostic information beyond implant position alone.

An additional sensitivity analysis provided important context for the observed association between valgus alignment and postoperative mechanical failure. Although valgus alignment was associated with an increased risk of mechanical failure in the primary exploratory model, this association was markedly attenuated and no longer statistically significant after additional adjustment for postoperative reduction-quality parameters, including calcar displacement, fragment displacement and angulation. Similar findings were observed when Baumgaertner reduction quality was included instead of its individual components. These findings suggest that valgus alignment itself is unlikely to represent an independent biomechanical risk factor but rather serves as a surrogate marker of poorer overall fracture reduction. Consequently, our findings further support the concept that postoperative reduction quality, rather than alignment alone, is the principal determinant of mechanical failure.

The findings of the present study have several practical implications for the surgical treatment of trochanteric femoral fractures. First, implant type was associated with the risk of revision surgery even within the same cephalomedullary nail platform; however, the observational study design precludes causal inference regarding implant selection. Our results also emphasize that meticulous fracture reduction remains substantially more important than implant choice itself. Restoration of calcar support, minimization of postoperative fragment displacement and achievement of a good Baumgaertner reduction should therefore remain primary surgical objectives. These parameters represent modifiable intraoperative factors that may reduce the risk of postoperative complications and mechanical failure irrespective of the cephalic fixation element used. Future research should prioritize prospective multicenter studies with standardized follow-up to validate the observed association between implant type and revision risk within the TFNA platform. Such studies should incorporate systematic assessment of mortality as a competing event, standardized evaluation of reduction quality, and direct measures of bone quality to further clarify the relative contributions of implant design and modifiable surgical factors to mechanical failure.

This study has several strengths. To our knowledge, it represents one of the largest TFNA-specific comparative analyses evaluating helical blade and lag screw fixation within an identical cephalomedullary nail platform. Furthermore, the systematic assessment of detailed postoperative radiographic parameters and the use of multivariable regression analyses allowed a comprehensive evaluation of reduction quality while accounting for important demographic, fracture-related and radiographic confounders, thereby strengthening the validity of the observed associations.

Several limitations should be acknowledged. First, the retrospective single-center design introduces the potential for residual confounding and selection bias despite multivariable adjustment. Importantly, implant type was strongly associated with treatment period, as the two fixation strategies were used predominantly during consecutive time periods. Therefore, unmeasured temporal changes in surgical technique, surgeon experience, reduction strategy, postoperative management, patient selection, or thresholds for revision may have contributed to the observed between-group differences. Baseline comparability between the groups cannot fully exclude such temporal confounding. An additional sensitivity analysis accounting for continuous calendar time yielded a directionally consistent but imprecise and non-significant estimate, and residual temporal confounding therefore cannot be excluded. Second, the elderly study population resulted in incomplete radiographic follow-up for some patients, as routine postoperative follow-up examinations were not consistently available due to death, frailty or limited mobility. Baseline clinical and radiographic characteristics were not systematically collected for patients who did not meet the predefined follow-up criterion. Therefore, differences between included and non-included patients could not be formally assessed, and the potential for selection bias related to differential follow-up could not be quantified. In addition, mortality was not systematically recorded as a longitudinal outcome after inclusion in the primary cohort, precluding a formal competing-risk analysis with death as a competing event. Because death may preclude subsequent surgical revision, the Kaplan–Meier and Cox analyses should therefore be interpreted with this limitation in mind. Consequently, a larger study population and a higher number of outcome events might have been achieved with more complete follow-up. In addition, follow-up duration differed significantly between the treatment groups, which may have influenced the observed crude event rates. While the crude proportion of surgical revisions was significantly higher in the blade group, this difference was not confirmed in time-to-event analyses accounting for censoring and unequal follow-up duration. Kaplan–Meier analysis, adjusted Cox regression, and fixed-time sensitivity analyses at 90 and 180 days consistently showed numerically lower revision-free survival after blade fixation, but no statistically significant between-group difference. Third, radiographic measurements were performed retrospectively, and image quality was not uniform across all examinations. Variations in projection and image quality occasionally limited the accuracy of certain measurements or precluded the assessment of specific radiographic parameters. Finally, although standardized measurement methods were applied, interobserver reliability was not formally assessed, which may have influenced the reproducibility of some radiographic parameters.

## 5. Conclusions

TFNA helical blade fixation was associated with a higher observed risk of surgical revision in the primary analysis (adjusted RR 1.84, 95% CI 1.07–3.15; *p* = 0.027); however, this association was not confirmed in time-to-event analyses or in the calendar-time-adjusted sensitivity analysis. Overall postoperative complication rates were comparable between groups (23.5% vs. 18.4%; *p* = 0.306). Given the retrospective, non-randomized study design, these findings should be interpreted as associations and do not establish a causal effect of implant type. Postoperative reduction quality, particularly residual calcar displacement, fragment displacement and Baumgaertner reduction quality, was consistently associated with postoperative complications and mechanical failure. These findings emphasize the importance of meticulous fracture reduction and highlight the need for further prospective multicenter studies with standardized follow-up.

## Figures and Tables

**Table 1:** Baseline demographic and clinical characteristics of the study cohort.

Variable	Overall (n = 277)	Blade (n = 136)	Screw (n = 141)	*p*-Value
Age, years	80.6 ± 10.7	81.3 ± 10.1	79.9 ± 11.3	0.275
Sex				0.147
Female	196 (70.8)	102 (75.0)	94 (66.7)	
Male	81 (29.2)	34 (25.0)	47 (33.3)	
Body mass index, kg/m^2^	23.1 ± 5.0	22.9 ± 5.2	23.3 ± 4.8	0.543
Osteoporosis	120 (43.3)	55 (40.4)	65 (46.1)	0.396
ASA class				0.250
ASA I	1 (0.4)	0 (0.0)	1 (0.7)	
ASA II	66 (23.8)	34 (25.0)	32 (22.7)	
ASA III	193 (69.7)	97 (71.3)	96 (68.1)	
ASA IV	17 (6.1)	5 (3.7)	12 (8.5)	
Pre-existing hip OA grade *				0.070
Grade 0	15 (5.4)	4 (2.9)	11 (7.8)	
Grade 1	97 (35.0)	52 (38.2)	45 (31.9)	
Grade 2	101 (36.5)	55 (40.4)	46 (32.6)	
Grade 3	55 (19.9)	23 (16.9)	32 (22.7)	
Grade 4	9 (3.2)	2 (1.5)	7 (5.0)	
Cognitive impairment	77 (27.9)	38 (27.9)	39 (27.9)	>0.999
Preoperative mobility				0.229
Independent	213 (77.7)	110 (82.1)	103 (73.6)	
Walking aid	57 (20.8)	23 (17.2)	34 (24.3)	
Wheelchair/bedbound	4 (1.5)	1 (0.7)	3 (2.1)	
Not reported	3	2	1	
Time from accident to surgery, days	1 (0–31)	1 (0–8)	1 (0–31)	0.294

Values are presented as mean ± standard deviation or as number (percentage). Time from accident to surgery is reported as median (range). * Radiographic hip osteoarthritis was graded according to the Kellgren–Lawrence classification. All *p*-values represent comparisons between the blade and screw groups. ASA, American Society of Anesthesiologists; OA, osteoarthritis.

**Table 2:** Injury characteristics, fracture morphology, femoral geometry and operative parameters.

Variable	Overall (n = 277)	Blade (n = 136)	Screw (n = 141)	*p*-Value
Type of accident				0.144
Fall	240 (86.6)	124 (91.2)	116 (82.3)	
Bicycle/e-bike/motorcycle	7 (2.5)	4 (2.9)	3 (2.1)	
Dizziness/syncope	12 (4.3)	3 (2.2)	9 (6.4)	
Unobserved fall	11 (4.0)	3 (2.2)	8 (5.7)	
Other	7 (2.5)	2 (1.5)	5 (3.5)	
AO/OTA classification				0.164
31A1.1	19 (6.9)	10 (7.4)	9 (6.4)	
31A1.2	66 (23.8)	34 (25.0)	32 (22.7)	
31A1.3	32 (11.6)	15 (11.0)	17 (12.1)	
31A2.1	35 (12.6)	21 (15.4)	14 (9.9)	
31A2.2	55 (19.9)	27 (19.9)	28 (19.9)	
31A2.3	30 (10.8)	11 (8.1)	19 (13.5)	
31A3.1	10 (3.6)	7 (5.1)	3 (2.1)	
31A3.2	5 (1.8)	4 (2.9)	1 (0.7)	
31A3.3	25 (9.0)	7 (5.1)	18 (12.8)	
Cortical thickness index, fracture side	0.48 ± 0.09	0.48 ± 0.10	0.48 ± 0.09	0.821
Cortical thickness index, contralateral side	0.53 ± 0.08	0.53 ± 0.07	0.53 ± 0.08	0.997
Cement augmentation	8 (2.9)	2 (1.5)	6 (4.3)	0.283
Additional cerclage	39 (14.1)	18 (13.2)	21 (14.9)	0.732
Tip–apex distance, mm	21.2 ± 7.6	20.8 ± 8.4	21.6 ± 6.8	0.432
Postoperative alignment				0.590
Anatomical	145 (52.7)	67 (49.6)	78 (55.7)	
Varus	76 (27.6)	39 (28.9)	37 (26.4)	
Valgus	54 (19.6)	29 (21.5)	25 (17.9)	
Not reported	2	1	1	
Calcar displacement, AP				0.732
Neutral	197 (71.6)	96 (71.1)	101 (72.1)	
Medial	59 (21.5)	31 (23.0)	28 (20.0)	
Lateral	19 (6.9)	8 (5.9)	11 (7.9)	
Not reported	2	1	1	
Calcar displacement, lateral				0.690
Neutral	132 (48.2)	66 (49.3)	66 (47.1)	
Anterior	40 (14.6)	17 (12.7)	23 (16.4)	
Posterior	102 (37.2)	51 (38.1)	51 (36.4)	
Not reported	3	2	1	
Fragment displacement, AP				0.648
<4 mm	223 (81.1)	111 (82.2)	112 (80.0)	
>4 mm	52 (18.9)	24 (17.8)	28 (20.0)	
Not reported	2	1	1	
Fragment displacement, lateral				0.194
<4 mm	189 (68.7)	98 (72.6)	91 (65.0)	
>4 mm	86 (31.3)	37 (27.4)	49 (35.0)	
Not reported	2	1	1	
Fracture angulation				0.775
<20°	211 (77.0)	102 (76.1)	109 (77.9)	
≥20°	63 (23.0)	32 (23.9)	31 (22.1)	
Not reported	3	2	1	
Baumgaertner reduction quality				0.088
Good	94 (34.3)	43 (32.1)	51 (36.4)	
Acceptable	128 (46.7)	71 (53.0)	57 (40.7)	
Poor	52 (19.0)	20 (14.9)	32 (22.9)	
Not reported	3	2	1	
Blade/screw tip position				**0.044**
Central	115 (41.5)	68 (50.0)	47 (33.3)	
Central–anterior	6 (2.2)	3 (2.2)	3 (2.1)	
Central–posterior	6 (2.2)	1 (0.7)	5 (3.5)	
Central–cranial	2 (0.7)	0 (0.0)	2 (1.4)	
Central–caudal	2 (0.7)	0 (0.0)	2 (1.4)	
Decentralized–anterior	21 (7.6)	9 (6.6)	12 (8.5)	
Decentralized–posterior	9 (3.2)	4 (2.9)	5 (3.5)	
Decentralized–cranial	25 (9.0)	7 (5.1)	18 (12.8)	
Decentralized–caudal	19 (6.9)	11 (8.1)	8 (5.7)	
Decentralized–cranial–anterior	32 (11.6)	14 (10.3)	18 (12.8)	
Decentralized–cranial–posterior	12 (4.3)	3 (2.2)	9 (6.4)	
Decentralized–caudal–anterior	19 (6.9)	12 (8.8)	7 (5.0)	
Decentralized–caudal–posterior	9 (3.2)	4 (2.9)	5 (3.5)	

Values are reported as mean ± standard deviation or as number (percentage). All *p*-values represent comparisons between the blade and screw groups. Statistically significant *p*-values (*p* < 0.05) are shown in bold. AO/OTA, Arbeitsgemeinschaft für Osteosynthesefragen/Orthopaedic Trauma Association; AP, anteroposterior.

**Table 3:** Complications, revision procedures and short-term outcomes according to implant group.

Complication or Outcome	Overall (n = 277)	Blade (n = 136)	Screw (n = 141)	*p*-Value
Any intraoperative complication	8 (2.9)	4 (2.9)	4 (2.8)	>0.999
Lateral wall fracture	1 (0.4)	0 (0.0)	1 (0.7)	>0.999
Cut-through	2 (0.7)	0 (0.0)	2 (1.4)	0.498
Cut-out	2 (0.7)	2 (1.5)	0 (0.0)	0.240
Greater trochanter dislocation	1 (0.4)	1 (0.7)	0 (0.0)	0.491
Near perforation	1 (0.4)	1 (0.7)	0 (0.0)	0.491
Any postoperative complication	58 (20.9)	32 (23.5)	26 (18.4)	0.306
Cut-out	22 (7.9)	15 (11.0)	7 (5.0)	0.076
Cut-through	4 (1.4)	1 (0.7)	3 (2.1)	0.623
Cut-in	6 (2.2)	6 (4.4)	0 (0.0)	**0.013**
Lateralization of neck implant	5 (1.8)	0 (0.0)	5 (3.5)	0.060
Non-union during follow-up	2 (0.7)	1 (0.7)	1 (0.7)	>0.999
Peri-implant infection	2 (0.7)	0 (0.0)	2 (1.4)	0.498
Peri-implant fracture	8 (2.9)	2 (1.5)	6 (4.3)	0.283
Impending cut-out	3 (1.1)	3 (2.2)	0 (0.0)	0.117
Fragment displacement	1 (0.4)	1 (0.7)	0 (0.0)	>0.999
Bolt breakage	1 (0.4)	0 (0.0)	1 (0.7)	>0.999
Implant breakage	1 (0.4)	0 (0.0)	1 (0.7)	>0.999
Symptomatic hardware	1 (0.4)	1 (0.7)	0 (0.0)	0.491
Any surgical revision	47 (17.0)	30 (22.1)	17 (12.1)	**0.037**
Revision to blade	2 (0.7)	2 (1.5)	0 (0.0)	0.240
Revision to screw	10 (3.6)	9 (6.6)	1 (0.7)	**0.009**
Conversion to total hip arthroplasty	21 (7.6)	14 (10.3)	7 (5.0)	0.114
Revision with HWR	1 (0.4)	0 (0.0)	1 (0.7)	>0.999
Revision to long TFNA	9 (3.2)	2 (1.5)	7 (5.0)	0.173
Revision to plate osteosynthesis	3 (1.1)	2 (1.5)	1 (0.7)	0.617
HWR	1 (0.4)	1 (0.7)	0 (0.0)	0.491
Discharge destination				0.305
Rehabilitation clinic	76 (27.4)	35 (25.7)	41 (29.1)	
Temporary nursing home	14 (5.1)	7 (5.1)	7 (5.0)	
Home	25 (9.0)	17 (12.5)	8 (5.7)	
Geriatric ward	121 (43.7)	55 (40.4)	66 (46.8)	
Nursing home	41 (14.8)	22 (16.2)	19 (13.5)	

Values are reported as number (percentage). Individual complication and revision subtypes were considered exploratory. Raw *p*-values are shown in the table. For individual postoperative complication comparisons, multiplicity was additionally assessed using Holm and Benjamini–Hochberg corrections; the unadjusted cut-in finding (*p* = 0.013) was not statistically significant after either correction (adjusted *p* = 0.146). All *p*-values represent comparisons between the blade and screw groups. Statistically significant raw *p*-values (*p* < 0.05) are shown in bold. HWR, hardware removal; TFNA, Trochanteric Femoral Nail Advanced.

**Table 4:** Adjusted risk ratios for intraoperative complications, postoperative complications and surgical revision according to bone and surgical characteristics.

Outcome and Predictor	Adjusted RR (95% CI)	*p*-Value
***Any intraoperative complication***		
CTI, fracture side	0.860 (0.338–2.189)	0.752
CTI, contralateral side	1.403 (0.490–4.020)	0.529
***Any postoperative complication***		
AO/OTA A2 vs. A1	1.061 (0.637–1.767)	0.819
AO/OTA A3 vs. A1	1.292 (0.671–2.487)	0.444
CTI, fracture side	0.862 (0.668–1.113)	0.255
Tip–apex distance	0.987 (0.842–1.157)	0.873
CTI, contralateral side	1.165 (0.849–1.600)	0.344
Varus vs. anatomical alignment	1.347 (0.786–2.306)	0.278
Valgus vs. anatomical alignment	1.461 (0.805–2.652)	0.212
Medial vs. neutral calcar displacement, AP	2.308 (1.435–3.710)	**<0.001**
Lateral vs. neutral calcar displacement, AP	2.126 (1.013–4.459)	**0.046**
Anterior vs. neutral calcar displacement, lateral	2.238 (1.249–4.010)	**0.007**
Posterior vs. neutral calcar displacement, lateral	1.356 (0.787–2.335)	0.273
Fragment displacement > 4 vs. <4 mm, AP	2.522 (1.613–3.943)	**<0.001**
Fragment displacement > 4 vs. <4 mm, lateral	1.522 (0.949–2.442)	0.082
Angulation ≥ 20° vs. <20°	1.423 (0.862–2.349)	0.168
Acceptable vs. good reduction	1.651 (0.883–3.086)	0.116
Poor vs. good reduction	2.626 (1.353–5.097)	**0.004**
Decentralized vs. central tip position	1.205 (0.750–1.935)	0.441
***Any surgical revision***		
AO/OTA A2 vs. A1	1.402 (0.777–2.529)	0.261
AO/OTA A3 vs. A1	1.512 (0.707–3.231)	0.286
CTI, fracture side	0.890 (0.662–1.198)	0.443
Tip–apex distance	1.037 (0.886–1.214)	0.650
CTI, contralateral side	1.219 (0.831–1.787)	0.312
Varus vs. anatomical alignment	1.298 (0.718–2.345)	0.388
Valgus vs. anatomical alignment	1.328 (0.675–2.614)	0.412
Medial vs. neutral calcar displacement, AP	2.334 (1.385–3.932)	**0.001**
Lateral vs. neutral calcar displacement, AP	1.711 (0.686–4.272)	0.250
Anterior vs. neutral calcar displacement, lateral	2.574 (1.318–5.026)	**0.006**
Posterior vs. neutral calcar displacement, lateral	1.530 (0.831–2.819)	0.172
Fragment displacement > 4 vs. <4 mm, AP	2.973 (1.809–4.886)	**<0.001**
Fragment displacement > 4 vs. <4 mm, lateral	1.709 (1.009–2.895)	**0.046**
Angulation ≥ 20° vs. <20°	1.016 (0.546–1.892)	0.959
Acceptable vs. good reduction	2.238 (1.060–4.723)	**0.035**
Poor vs. good reduction	3.049 (1.356–6.856)	**0.007**
Decentralized vs. central tip position	1.279 (0.751–2.177)	0.366

Each predictor was adjusted for implant type, age and sex. Reference categories were AO/OTA A1, anatomical alignment, neutral calcar position, fragment displacement < 4 mm, angulation < 20°, good Baumgaertner reduction quality and central tip position. Distance effects are reported per 5-mm increase. Statistically significant *p*-values (*p* < 0.05) are shown in bold. AP, anteroposterior; CI, confidence interval; CTI, cortical thickness index; RR, risk ratio.

**Table 5:** Adjusted risk factors for postoperative mechanical failure.

Predictor	Adjusted RR (95% CI)	*p*-Value
AO/OTA A2 vs. A1	1.233 (0.603–2.521)	0.566
AO/OTA A3 vs. A1	1.251 (0.487–3.217)	0.642
CTI, fracture side	0.886 (0.598–1.312)	0.545
Tip–apex distance, per 5 mm increase	1.090 (0.882–1.347)	0.425
CTI, contralateral side	1.211 (0.759–1.934)	0.422
Varus vs. anatomical alignment	1.250 (0.578–2.706)	0.570
Valgus vs. anatomical alignment	2.352 (1.073–5.155)	**0.033**
Medial vs. neutral calcar displacement, AP	3.612 (1.854–7.037)	**<0.001**
Lateral vs. neutral calcar displacement, AP	3.034 (1.121–8.213)	**0.029**
Anterior vs. neutral calcar displacement, lateral	3.016 (1.180–7.713)	**0.021**
Posterior vs. neutral calcar displacement, lateral	2.636 (1.186–5.856)	**0.017**
Fragment displacement > 4 vs. <4 mm, AP	4.027 (2.189–7.408)	**<0.001**
Fragment displacement > 4 vs. <4 mm, lateral	2.950 (1.539–5.655)	**0.001**
Angulation ≥ 20° vs. <20°	1.736 (0.880–3.423)	0.111
Acceptable vs. good reduction	3.772 (1.151–12.364)	**0.028**
Poor vs. good reduction	6.921 (1.953–24.527)	**0.003**
Decentralized vs. central tip position	1.751 (0.865–3.543)	0.119

Mechanical implant failure was defined as cut-out, cut-through or cut-in. Each predictor was adjusted for implant type, age and sex. Tip–apex distance effects are reported per 5 mm increase; CTI effects are reported per 0.1-unit increase. Reference categories were AO/OTA A1, anatomical alignment, neutral calcar displacement, fragment displacement < 4 mm, angulation < 20°, good Baumgaertner reduction quality and central blade/screw tip position. Statistically significant *p*-values (*p* < 0.05) are shown in bold. AP, anteroposterior; CI, confidence interval; CTI, cortical thickness index; RR, risk ratio.

**Table 6:** Sensitivity analyses evaluating the association between postoperative alignment and mechanical failure.

**Panel A. Adjusted risk ratios according to postoperative alignment.**					
**Model**	**Alignment**	**N**	**Events**	**Adjusted RR (95% CI)**	***p*** **-Value**
Core adjusted model	Varus vs. anatomical	250	30	1.344 (0.564–3.200)	0.505
Core adjusted model	Valgus vs. anatomical	250	30	2.773 (1.181–6.512)	**0.019**
Reduction-component model	Varus vs. anatomical	248	30	0.790 (0.307–2.030)	0.624
Reduction-component model	Valgus vs. anatomical	248	30	1.275 (0.422–3.849)	0.666
Baumgaertner model	Varus vs. anatomical	249	30	0.673 (0.254–1.785)	0.427
Baumgaertner model	Valgus vs. anatomical	249	30	2.182 (0.854–5.573)	0.103
**Panel B. Standardized adjusted absolute risks according to postoperative alignment.**					
**Model**	**Alignment**	**Adjusted Mechanical-Failure risk, %**		**Adjusted RR vs. Anatomical**	
Core adjusted model	Anatomical	8.3		1.000	
Core adjusted model	Varus	11.1		1.344	
Core adjusted model	Valgus	22.9		2.773	
Reduction-component model	Anatomical	12.1		1.000	
Reduction-component model	Varus	9.6		0.790	
Reduction-component model	Valgus	15.4		1.275	
Baumgaertner model	Anatomical	11.4		1.000	
Baumgaertner model	Varus	7.7		0.673	
Baumgaertner model	Valgus	24.9		2.182	
**Panel C. Direct comparison between valgus and varus alignment.**					
**Model**	**Comparison**	**N**	**Events**	**Adjusted RR (95% CI)**	***p*** **-Value**
Core adjusted model	Valgus vs. varus	250	30	2.064 (0.888–4.796)	0.092
Reduction-component model	Valgus vs. varus	248	30	1.615 (0.633–4.119)	0.316
Baumgaertner model	Valgus vs. varus	249	30	3.240 (1.357–7.739)	**0.008**

Modified Poisson regression with robust standard errors. The core model adjusted for implant type, age, sex, AO/OTA classification, fracture-side CTI, contralateral CTI, tip–apex distance and tip position. The reduction-component model additionally adjusted for calcar displacement, fragment displacement and angulation. The Baumgaertner model included Baumgaertner reduction quality instead of its individual components. Statistically significant *p*-values (*p* < 0.05) are shown in bold. CI, confidence interval; CTI, cortical thickness index; RR, risk ratio.

**Table 7:** Cox model adjusted for age, sex, osteoporosis.

Variable	Adjusted HR (95% CI)	*p*-Value
Implant type: Blade vs. Screw	1.46 (0.78–2.73)	0.240
Age, per 1-year increase	1.01 (0.98–1.04)	0.559
Sex: Female vs. Male	1.08 (0.53–2.18)	0.835
Osteoporosis: Yes vs. No	1.34 (0.74–2.42)	0.328

Cox proportional hazards regression evaluating implant type as the exposure of interest and adjusted for age, sex, and osteoporosis. Screw fixation, male sex, and absence of osteoporosis were the reference categories. Patients without revision were censored at the last documented radiographic follow-up. CI, confidence interval; HR, hazard ratio.

**Table 8:** Follow-up duration according to implant type.

Variable	Overall (n = 277)	Blade (n = 136)	Screw (n = 141)	*p*-Value
Follow-up duration, days	231.0 ± 173.0	299.2 ± 203.5	165.2 ± 100.9	**<0.001**

Values are presented as mean ± standard deviation and were compared using Welch’s independent-samples *t*-test. Statistically significant *p*-values (*p* < 0.05) are shown in bold.

## Data Availability

The data presented in this study are available on reasonable request from the corresponding author. The data are not publicly available because they contain information that could compromise the privacy of research participants.

## Abbreviations

The following abbreviations are used in this manuscript:

AO/OTA	Arbeitsgemeinschaft für Osteosynthesefragen/Orthopaedic Trauma Association
AP	Anteroposterior
ASA	American Society of Anesthesiologists
BMI	Body mass index
CI	Confidence interval
CTI	Cortical thickness index
HR	Hazard ratio
HWR	Hardware removal
IQR	Interquartile range
OA	Osteoarthritis
PACS	Picture archiving and communication system
RR	Risk ratio
SD	Standard deviation
STROBE	Strengthening the Reporting of Observational Studies in Epidemiology
TAD	Tip–apex distance
TFNA	Trochanteric Femoral Nail Advanced
THA	Total hip arthroplasty
